# The Potential Use of Isothermal Amplification Assays for In-Field Diagnostics of Plant Pathogens

**DOI:** 10.3390/plants10112424

**Published:** 2021-11-10

**Authors:** Aleksandr V. Ivanov, Irina V. Safenkova, Anatoly V. Zherdev, Boris B. Dzantiev

**Affiliations:** A.N. Bach Institute of Biochemistry, Research Centre of Biotechnology, Russian Academy of Sciences, Leninsky Prospect 33, 119071 Moscow, Russia; a.ivanov@fbras.ru (A.V.I.); safenkova@inbi.ras.ru (I.V.S.); zherdev@inbi.ras.ru (A.V.Z.)

**Keywords:** out-of-lab diagnostics, molecular diagnostics, plant diseases, isothermal amplification, DNA amplicon detection, recombinase polymerase amplification, loop-mediated amplification, rolling circle amplification, nucleic acid sequence-based amplification, helicase dependent amplification

## Abstract

Rapid, sensitive, and timely diagnostics are essential for protecting plants from pathogens. Commonly, PCR techniques are used in laboratories for highly sensitive detection of DNA/RNA from viral, viroid, bacterial, and fungal pathogens of plants. However, using PCR-based methods for in-field diagnostics is a challenge and sometimes nearly impossible. With the advent of isothermal amplification methods, which provide amplification of nucleic acids at a certain temperature and do not require thermocyclic equipment, going beyond the laboratory has become a reality for molecular diagnostics. The amplification stage ceases to be limited by time and instruments. Challenges to solve involve finding suitable approaches for rapid and user-friendly plant preparation and detection of amplicons after amplification. Here, we summarize approaches for in-field diagnostics of phytopathogens based on different types of isothermal amplification and discuss their advantages and disadvantages. In this review, we consider a combination of isothermal amplification methods with extraction and detection methods compatible with in-field phytodiagnostics. Molecular diagnostics in out-of-lab conditions are of particular importance for protecting against viral, bacterial, and fungal phytopathogens in order to quickly prevent and control the spread of disease. We believe that the development of rapid, sensitive, and equipment-free nucleic acid detection methods is the future of phytodiagnostics, and its benefits are already visible.

## 1. Introduction

Global agriculture faces serious harm through the spread of phytopathogens. Recently, the spread of pathogens has become a particularly urgent issue owing to two important factors of the modern world. First, active international trade and plant imports by a highly mobile modern society have led to the transfer of phytopathogens to new regions [1,2]. Second, climate change is influencing changes in the resistance of certain plant species to pathogens, the transformation of host–pathogen relations, and the severity of diseases [3]. Pathogen infection can start from seed material and accumulate in a latent form for several generations before abruptly manifesting through suitable environmental conditions, causing a significant reduction in the quality of cultivated products and subsequent serious losses in yield [4,5,6].

Disease control involves several principles: avoidance, exclusion, eradication, protection, resistance, and therapy [7]. National and international legislations for control of quarantine pathogens aims to prevent outbreaks at the inoculum stage [8,9]. Another control strategy is to prevent infection spread by detecting pathogens in wild and/or cultivated populations [10,11]. Different therapy approaches include: chemical treatment (especially fungicides) [12,13], peptides [14], bacteriophages [15], infection by non-pathogenic competitors of a pathogen [16], and cryotherapy [17]. In addition, conventional breeding for resistance cultivars and the development of transgenic plants limits pathogen attacks and builds resistance in crops and plants [18]. However, the most cost-effective and feasible strategy is early detection and prevention to control the pathogen and reduce yield damage. A suitable way to do this is through the application of rapid diagnostic tools with high specificity and sensitivity. Using such tools for pathogen detection should be simple and accessible and ultimately be able to be used in different locations such as laboratories, in-field, a certified diseases control centre, at borders, a local farm, or a greenhouse. For each situation or site, different but adequate tools for diagnostics may be required [19].

Diagnosing plant diseases by visually assessing symptoms often does not provide a comprehensive assessment. Some infections demonstrate mild or nonspecific symptoms or be asymptomatic and thus require a verified diagnostic test to determine the pathogen(s) causing the disease [20]. Many methods exist for detecting different plant pathogens. These can be divided into three types: taxonomical identification of pathogens using microbiological approaches, immunoassays based on antigen detection (proteins, peptides, and polysaccharides), and detection of the nucleic acids of a pathogen [20,21]. Microbiological approaches are extremely time-consuming and require highly qualified staff to perform the analysis. There are many immunoassay-based approaches, including rapid (5–10 min) assays; however, the sensitivity of these methods is largely limited by the affinity of the antigen–antibody interaction: it is not always possible to use a high-affinity antibody. In addition, the specificity of immunoreagents cannot always ensure the absence of cross-reactivity. Assays based on the amplification and detection of nucleic acids, provides a targeted, highly sensitivity diagnostic tool for the identification of phytopathogens [22]. Some approaches even allow for the detection of a single nucleotide substitution [23]. The most conventional amplification method is PCR, the gold standard for nucleic acid detection. However, PCR-based methods can be time-consuming and are difficult to perform outside of the laboratory due to the limitation of heavy equipment that ensures cyclic reactions are carried out at different temperatures. PCR assays can be actively used in laboratories but are not suitable as field-deployment assays at field sites, customs, or farms.

Today, alongside the traditional approaches based on the detection of nucleic acids, there are isothermal amplification methods [24]. The main advantage of isothermal amplification is the ability to amplify RNA/DNA at a constant temperature, thus enabling the use of less complex equipment. There are many methods, such as recombinase polymerase amplification (RPA), loop-mediated amplification (LAMP), helicase-dependent amplification (HDA), nucleic acid sequence-based amplification (NASBA), and rolling circle amplification (RCA) [24]. All these methods have different amplification temperatures, sensitivities, reaction times, and other pros and cons. For all of these, the amplification stage ceases to be limited by time and instruments. Thus, the important task is to find appropriate approaches for rapid and non-device preparation of plant samples and equipment-free detection of amplicons after amplification. By equipment-free detection after amplification, we mean the detection of a signal with the naked eye without specialized devices or tools. Visualization of a signal after amplification can be achieved by changes in colour of certain compounds in the presence of DNA, fluorescence detection using only a UV lamp, nanoparticle (NP) aggregation, binding of labelled DNA fragments on a membrane or test strips, or specific hybridization assays.

**Figure 1:**
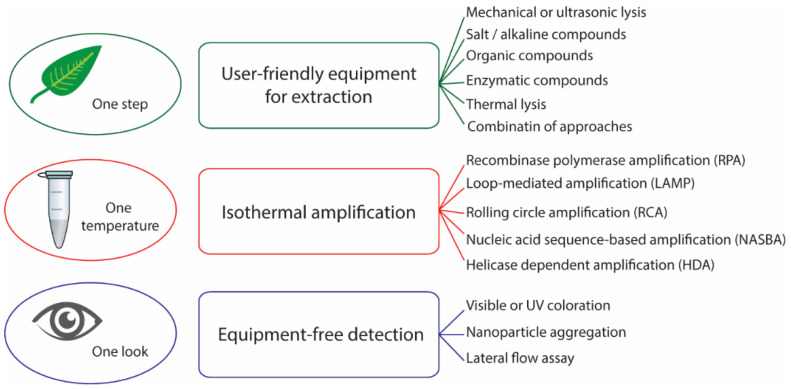
The main kinds of extraction, isothermal amplification, and amplicon detection for rapid, sensitive, and in-field diagnostics testing of viral and other plant pathogens.

In this review, we consider the detection of plant pathogens through a combination of isothermal amplification methods with extraction and detection approaches compatible with in-field diagnostics (Figure 1). We summarize the main trends in pathogen RNA/DNA genomic extraction, including homogenization of plant tissues and discuss possible advantages and limitations for different in-field extraction methods. We collected and compared data on the sensitivity of the isothermal amplification methods and compared different approaches to amplification and detection by their complexity, sensitivity, and tolerance to inhibitors. We review different methods of DNA amplicon visualization and discuss their suitability for simple and rapid in-field diagnostics. The review provides a large number of examples for the detection of viral, bacterial, and other types of phytopathogens.

## 2. Extraction of Pathogen Genome from Plants

The first stage of the assay is to extract the pathogen genome from plants. To select an appropriate sample preparation method, it is necessary to understand the localization of the pathogen in the organs and tissue of the plants and the plant components that inhibit the amplification reaction, which must be removed beforehand. Most pathogens have a referenceable plant organ as a target, although pathogens can be transported within plants [20]. Plant viruses are characterized by the presence of RNA, whereas DNA is less common. Therefore, plant viruses and viroid’s have proposed protocols for RNA extraction, while other pathogens have proposed protocols for DNA extraction.

Consideration must be given to the plants’ structural features, organs and tissues (cellulose cell walls and fibres) for the extraction of both plant and pathogen genomes. Thus, the first step of any extraction method is homogenization of plant tissue in appropriate solutions, and the second, is to destroy cellulose fibres, followed by the capturing and purification of nucleic acids from the sample.

### 2.1. Pre-Treatment of Plant Tissue for Nucleic Acid Extraction

Pre-treating plant tissue is crucial for rapid diagnostics. Accessibility of DNA/RNA for subsequent extraction and purification is limited by the effectiveness of tissue homogenization. All DNA/RNA extraction methods require a liquid state when nucleic acids are soluble. The method of homogenization depends on the stiffness of the plant tissues. Soft leaves and crops or sap can be homogenized without much mechanical treatment, by shaking in appropriate buffer and can be performed at any site [25,26]. Soft parts of a plant can be macerated by plastic mesh in a zip-lock bag (e.g., Sample Extraction Bag 1, Agdia) in appropriate buffer [27], even in field conditions. Use of a hand-powered mortar and pestle allows the maceration of samples with greater stiffness. Milling by metallic beads can also homogenize medium-firm samples and can be easily automized [28]. Rotor-grinding devices with blades such as food processors can homogenize even firm samples. The available grinding devices include portable models with disposable sample-grinding containers (see Dremel, Robert Bosch Gmbh, as an example) [29]. Homogenization of lignified tissue from wood samples is the most difficult to pre-treat. Often, it requires an additional step before grinding, such as shredding of hard tissue to a powder. Drilling wood cross-sections allows sawdust powder to be obtained for resuspension in buffer before DNA extraction [30,31,32]. Similarly, maceration of sawdust in a mortar facilitates the subsequent homogenization [33]. Wood tissue shavings obtained by a scalpel, followed by maceration, may be used as a substitute for drilling [34,35]. However, these tools are fit only for sawn-off fragments. Bark grinding with homemade [36] or commercial [29] tools make it possible to test living trees. An increment borer enables wood samples to be obtained from living trees which can be homogenized by grinders or mortars [37,38]. Non-invasive collection of samples (wood chips or frass) can also be used for subsequent grinding and DNA extraction [39,40]. Therefore, various convenient tools are available and can be used for pre-treating different plant tissues in the field.

### 2.2. Cell Disruption and Assessment of Inhibition Resistance for Isothermal Amplifications

Cellular lysis is necessary for releasing pathogen DNA/RNA from plant cells. Effective pre-treatments can cause cell wall destruction and release some DNA/RNA in the homogenate. This crude extract can be used for in-field diagnostics by RPA or LAMP without DNA/RNA purification. However, an additional stage of cell wall disruption increases DNA/RNA yield dramatically. Enzymatic cleavage of target nucleic acids during extraction should be minimized (e.g., EDTA and an RNAse inhibitor addition). Plant polysaccharides and other metabolites can affect downstream applications and should be removed [41]. PCR is sensitive to inhibitors such as polyphenols, tannic acid, pectin, and xylene [42,43]. However, information is scarce on inhibitors of isothermal reactions, so to compare the features of different isothermal approaches, we looked at examples concerning not only plant samples. LAMP polymerase BstI is more tolerant to some inhibitors than PCR [44,45,46], allowing LAMP to be used for the diagnosis of plant pathogens in crude extracts [47,48,49]. RPA manufacturers declare high tolerance of their systems to inhibitors; indeed, RPA was shown to demonstrate tolerance to plant inhibitors [50,51,52]. HDA utilizes BstI polymerase, similar to LAMP, so it should demonstrate similar inhibition tolerance, and to date there is no data on inhibition of HDA reactions by plant metabolites. Neither NASBA or RCA have been examined for plant inhibition tolerance. Some researchers of non-plant pathogens found NASBA to be more [53] or equally [54] sensitive to inhibitors than PCR. Background DNA/RNA can inhibit RPA reactions [55,56]. Birch et al. [57] showed that large concentrations of RNA inhibited NASBA reactions, and samples needed to be diluted. Therefore, an increase in the number of initial DNA/RNA targets, or dilution of samples, abrogates the DNA/RNA background inhibition effects. Additionally, HDA inhibition by background DNA was also demonstrated for non-plant pathogen detection [58].

### 2.3. Chemical Protocols for Homemade Extractions

Most plant organs can be extracted through a single protocol. The first proposed protocol, utilizing cetyltrimethylammonium bromide (CTAB) [59], is still popular and has had many modifications. The CTAB method is appropriate for DNA extraction from bacteria, fungi, plant, and animal tissues [60,61,62]. Moreover, RNA can be extracted through a modified CTAB method [63]. Briefly, a tissue sample is homogenized with a grinder or pestle. For cellular lysis, the homogenate is incubated at 60 °C in a high-salt buffer with CTAB as a detergent that provides different levels of solubility for DNA and polysaccharides. Furthermore, DNA extraction is performed with chloroform followed by ethanol or isopropanol precipitation. The advantage of this extraction method is a high yield of DNA/RNA, including genomic DNA. A CTAB extraction commonly takes about 1 h, but in different proposed protocols its duration varies from 40 min [64] to 6 h [60]. The extraction requires a mini-centrifuge and using harmful compounds such as chloroform, which makes this method difficult for in-field application.

Other DNA/RNA extraction methods include the guanidine method based on protein denaturation followed by RNA separation from polysaccharides by phenol–chloroform extraction and ethanol purification. This method can also be combined with CTAB [65]. Another popular method is alkaline extraction [66], which can be used for plant DNA extraction [67]. High-molecular-weight genomic plant DNA can be extracted by rapid alkaline-PEG lysis protocol [68]. This method utilizes 20 mM NaOH and 6% PEG200 for lysis of plant leaf homogenates. Commercial kits have been designed to use this method with modifications, such as RNAeasy Plant Mini Kit [69]. Acceleration of the extraction and avoidance of harmful compounds can be achieved by using different resins, such as cellulose and silica [63,70,71]. For the extraction of DNA/RNA from specific tissue (wood, roots, pines, etc.), many protocols have been developed.

It should be noted that some extraction reagents can affect the downstream amplification reactions. Insufficient washing of extracted DNA/RNA from phenol–chloroform extraction could cause the inhibition of any enzymatic reaction. Strong chaotropic agents such as guanidine chloride also inhibit reactions to induce protein denaturation. EDTA binds magnesium ions causing inhibition of polymerase reactions. Although both LAMP and RPA utilize relatively high magnesium concentrations, the presence of EDTA in samples can cause inhibition [45]. Detergents such as CTAB (2%) and SDS (0.05%) can inhibit RPA [72,73]. LAMP demonstrated no sensitivity inhibition in the presence of 3% Triton X100, but signal response was significantly lower [74]. Using pathogen RNA/DNA extraction approaches without inhibitors could help overcome the limits. One possible method utilizes ammonium trichloroacetate for rapid viral RNA extraction from plant tissues [75].

### 2.4. Ready-to-Use Solutions (Commercial Kits) for Extraction

**Figure 2:**
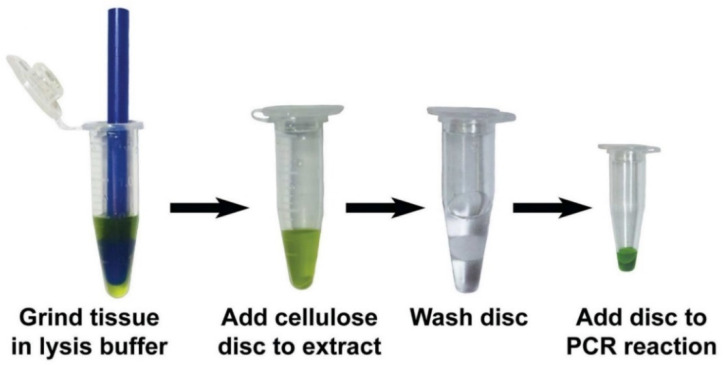
Rapid and simple approach for RNA extraction under 30 sec following mechanical pre-treatment of leaf, as proposed by Zou et al. [77]. Reprinted from [77] with permission from PLOS.

Rapid nucleic acid extraction using easily available and operated equipment is important for field or non-lab site diagnostics. The simplest means of achieving this is through mesh-bag homogenization in extraction buffer; however, this method does not purify DNA or RNA from cellular compounds that could inhibit downstream applications. Additionally, efficiency of extraction decreases using this method. The fastest extraction method is to use nitrocellulose lateral flow discs such as those produced by Abingdon Health (UK). The discs are incubated in homogenate for 2 min. A special buffer from the manufacturer provides absorption of DNA onto the discs, which are then dried for 5 min and can be used for amplification [76]. Another method is to use cellulose lateral flow dipsticks for absorption of DNA from the ground or macerated plant in a buffer with detergents. RNA extraction buffer contains guanidine hydrochloride and detergents. After washing the dipstick, elution of nucleic acids from the dipstick is performed directly into the amplification mix (Figure 2). This method can be performed in 30 s [77].

The requirement for effective and reproducible DNA/RNA extraction has led to the development of commercial kits for the extraction of nucleic acids from plant tissues. However, manufacturers do not disclose in detail the components of their kits. Some kits use organic solvents such as phenol and chloroform for lysis and phase separation, while others use alkaline or chaotropic agents. Samples with different origins often require a specific condition of lysis and washing to minimize contamination and provide maximal DNA/RNA retention. Manufacturers have considered this and produce kits for tissue-specific samples (plant, blood, soil, fungi, etc.). Most commercial kits are optimized for the separation of DNA or RNA from other cell compounds using gravity columns, spin columns containing anion-exchange material, or magnetic beads covered with DNA/RNA binding compounds (indicated in the “Separation” column of Table S1). Variations among the parameters of lysis, capturing, washing, and elution only enable the separation of RNA or DNA to be performed. Kits mentioned in the review are summarized in Table S1 (Comparison of commercial kits for nucleic acid extraction and purification). The majority of kits in Table S1 are proposed for use in equipped conditions as they need additional equipment such as devices for tissue grinding, heating, centrifuging, and vortexing. However, it is possible to have portable equipment for on-site (field) testing, such as the pre-treatment devices described above. Mini-centrifuges and other portable equipment were used successfully for in-field diagnostics of fungal infections [78]. Three-dimensional printing technology extends the possibilities by providing portable equipment for these purposes [79]. For example, a portable hand-powered centrifuge was printed and tested for DNA extraction from leaves in the forest [80]. It should be noted that only extraction procedures without the use of frozen plant tissues or harmful organic extractants (phenol or chloroform) can be adopted for in-field testing.

## 3. Isothermal Amplification

### 3.1. Recombinase Polymerase Amplification

**Figure 3:**
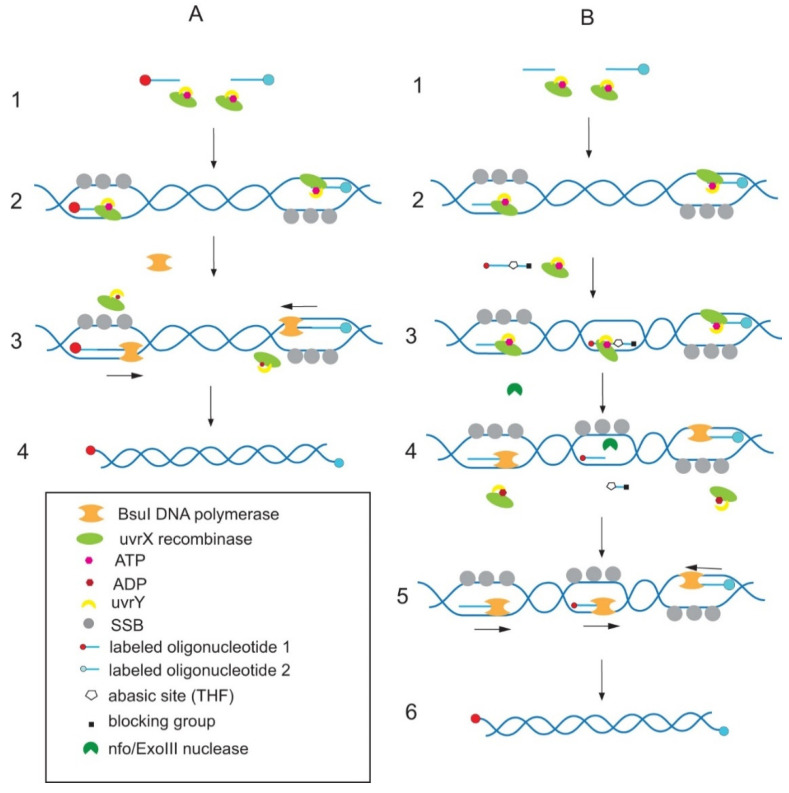
Scheme of recombinase polymerase amplification (RPA) **A**. Stages of basic RPA (TwistDx Basic): 1. Activation of primers by recombinase complex; 2. Recombinase-mediated primers annealing to target double-stranded (ds)DNA; 3. Recombinase complex dissociation and BsuI-dependent DNA polymerization; 4. Amplified DNA labelled by fluorescein (FAM) and biotin. **B**. Stages of RPA with probe (TwistDx nfo and TwistDx exo): 1. Activation of primers by recombinase complex; 2. Recombinase-mediated primers annealing to target dsDNA; 3. Activation of modified DNA probe by recombinase complex and recombinase-mediated probe annealing to target dsDNA between the primer annealing sites; 4. Dissociation of the recombinase complexes, start of polymerization from the primers, exonuclease III/nfo endonuclease IV dependent 5′–3′ digestion of probe from abasic site when the probe forms DNA duplex, dissociation of blocking fragment of the probe; 5. Polymerization of DNA from free 3′ of primers and probe; 6. Amplified DNA labelled by FAM and biotin.

Invented in 2006 [81], isothermal RPA is based on recombinase-dependent hybridization of primers with double-stranded (ds)DNA, instead of a temperature-dependent denaturation-annealing process during PCR. In the first stage, the primers form a complex with phage recombinase uvrX. The complex then binds with target dsDNA and displaces a DNA strand at the correct DNA site. This process requires a second additional recombinase loading factor through uvrY and ATP hydrolysis. The released single strand is stabilized by a single-strand binding (SSB) protein. BsuI DNA polymerase synthetizes DNA using these primers. The process of RPA is presented in Figure 3. The optimal length of primers is 28–35 nt for optimal complex formation. However, it is possible to use PCR-related primers. Typically, RPA synthesizes a mono-product with a length of 50–800 bp.

For rapid amplicon detection after RPA, two-sided labelled amplicons are most commonly used. There are two pathways to obtain labelled amplicons: either labelled forward and reverse primers or a one-labelled primer and an oligonucleotide labelled probe. The oligonucleotide probe comprises two oligonucleotides of different lengths connected with tetrahydrofuran (THF). A 5′ longer oligonucleotide is modified with fluorescein (FAM) and a 3′ shorter oligonucleotide is blocked. An annealing site for the probe must be chosen in the region between primer annealing sites. Having blocked the 3′ oligonucleotide, the probe cannot be used as a primer. However, the probe intercalates into dsDNA of amplified product in a recombinase-dependent manner. DNA-endonuclease Nfo or exonuclease ExoIII) cleaves THF abasic sites followed by the release of short oligonucleotides from the duplex. The cleaved labelled probe has a normal 3′ end and can be used as a primer. The new product contains FAM and biotin at the terminals of DNA molecules. The use of the second approach with the probe is intended to improve the specificity of the assay based on the labelled amplicon being formed only at double recognition sites (oligonucleotide probe and primer) [81].

The method has two main advantages, the reaction temperature and the duration time. The reaction proceeds at a temperature range of 30–42 °C, with the manufacturer recommending that the test be conducted at 39 °C. Moreover, RPA is tolerant to temperature fluctuations. The recommended time is 20 min, but more sensitive detection allows amplification within 10 min [82,83]. Additionally, RPA can be easily multiplexed for simultaneous detection of several pathogens [84]. Portable heating block provides the necessary temperature and can be applied for on-site testing. Moreover, some devices and reactions are being heated by body heat [84,85,86].

Despite the advantages of RPA, there are some specific drawbacks. RPA has a 1500 bp limitation on the length of the amplification product [81], and the manufacturer recommends designing the amplified product within 800 bp. The optimal length of the RPA DNA product for lateral flow assay (LFA) is 150 bp [87]. Another limitation of RPA is tolerance to mismatches in primers. RPA can give false positive results in cases where there are up to nine mismatches in both primers [88]. Moreover, the effect of mismatches is hardly predictable and depends on the GC-content of a primer, a number of mismatches, their position and distribution, and primer length. Even a mismatch at the 3′ terminus of each primer cannot provide total specificity [89]. Taken together, it shrinks the number of target sequences for detection in the genome of the organism of interest. Additionally, it reduces the possibility of discrimination of close-relative organisms. Another feature of RPA is the high viscosity of the reaction mix. It decreases diffusion and can affect the local amplification event and the amplification volume. These effects can interfere with amplification of low titre target DNA. The manufacturer recommends intensive shaking after 4 min of the reaction [90].

To date, RPA is the only isothermal method based on commercially developed kits for the detection of plant pathogens. Since 2012, RPA-based tests for different pathogens have been developed intensively. The RPA kit of TwistDx (Cambridge, UK) became commercially available for researchers, and at the same time, reverse transcription combined with RPA for RNA amplification was proposed [91]. More specialized commercial kits based on RPA have been designed for the detection of plant pathogens (plum pox virus, Candidatus Liberibacter asiaticus, grapevine red blotch-associated virus, Fusarium oxysporum f. sp. vasinfectum, Race 4, banana bunchy top virus, little cherry virus 2, tomato chlorotic dwarf viroid) as AmplifyRP^®^ (Agdia, Elkhart, IN, USA). These kits comprise simple equipment for homogenization (buffer, plastic bag, and mesh), lyophilized premix for RPA reaction with optimized primers and THF probe, and cassette with lateral flow test. In 2014, the first RT–RPA-based test was created for little cherry virus 2. RT–RPA was performed in crude plant extract followed by rapid in-field diagnostics. The sensitivity of these tests was 100 times lower than that of RT–qPCR, although this decreased sensitivity could be compensated for by rapid and easy analysis [92]. Now, there has been an increase in original papers on developing RPA tests for different plant pathogens. These articles will be discussed in detail in the context of their detection methods.

### 3.2. Loop-Mediated Amplification

LAMP refers to isothermal amplification based on the accurate design of four primers [93]. Unlike RPA, LAMP utilizes only the Bst polymerase enzyme at an optimum temperature of 60–65 °C. It typically takes 1 h to react. The enzyme has strand displacement activity that does not require high-temperature denaturation to be performed. A simpler LAMP method requires four primers: two loop generating primers and two primers necessary for the displacement of newly generated strands. After the first stage of amplification, the newly synthetized DNA molecule contains two terminal loop regions with a stem-loop region between them and resembles a dumbbell [93]. The dumbbell ssDNA is the initial unit for amplification, which is then extended to generate long-length products. The specific design of LAMP primers allows synthesis of many types of DNA products.

Modified LAMP involves two more primers annealing to these loops. They boost the reaction and synthesis more DNA products, accelerating the reaction by up to 30 min [94]. LAMP for RNA with reverse transcriptase has been developed [95]. The main advantage of LAMP is the low cost of the equipment and reaction compounds that are required. At the same time, precise primer design and the high number of primers for selection are the main obstacles for this analysis gaining traction. Nonoptimal primers and temperature generate nonspecific amplification and primer-dimer products [96]. Another drawback of LAMP is complex multiplexing due to the complexity of design of two or more sets of primers. That said, several multiplexed LAMP (for non-plant pathogens) systems have been developed [97].

There are many approaches for the detection of LAMP products, including dye staining for colouration, fluorescent labelling, lateral flow assay, electrochemistry detection, surface plasmon resonance, lab-on-chip, gold nanoparticles (GNP) aggregation caused by DNA binding, and pyrosequencing [98]. Examples that relate to the easy and equipment-free visualization of LAMP amplicons will be detailed in Section 4.1.2 and Section 4.3.2.

### 3.3. Rolling Circle Amplification

RCA was the first isothermal amplification method to be developed. Phi29 DNA polymerase binds to a duplex of circular ssDNA and a primer and synthesizes long ssDNA using its own strand displacement activity. The amplification generates multiple copies of circular DNA as repeated in linear ssDNA. The optimal temperature is 37 °C, and the duration is approximately 1 h [99]. Only some viruses have suitable DNA for this form of detection. Padlock-ligated probes have been proposed to overcome this obstacle [100]. A short ssDNA probe with a sequence along the 5′ and 3′ terminals that are complementary to the sequence of interest is hybridized with target dsDNA. The scheme of the complex looks like a padlock. After the complex forms, ligation of probe ends is performed by a ligase. The non-ligated probe should be removed by exonuclease. The next stage involves the RCA of the new circular ssDNA. The main weakness of this method is at the ligation stage. It can have low efficiency depending on substrate concentration or reaction conditions and be inhibited by some compounds in the sample. Nonetheless, RCA is a flexible platform for combination with various methods of enhancement, such as hyperbranching, multiple aptamer formation encoded in a padlock probe, and labelled probe annealing [101].

RCA with padlock ligation probes (PLP) and its modifications are used for plant pathogen detection. Some plant viruses (e.g., geminiviruses or nanoviruses) have circular ssDNA that can be an ideal target for RCA. However, most of the proposed tests are inappropriate for field diagnostics: gel electrophoresis, restriction endonuclease digestion assay, and deep sequencing of RCA products are applied for the detection and discrimination of different geminiviruses, such as Abutilon mosaic virus, the common strain of tomato golden mosaic virus, beet curly top virus, Sida golden mosaic Costa Rica virus, African cassava mosaic virus, and tomato golden mosaic virus-yellow vein [102]. Another research use for RCA–PLP is for the identification of geminiviruses (e.g., maize streak virus, Digitaria streak virus, Chloris striate mosaic virus, Miscanthus streak virus, wheat dwarf virus, barley dwarf virus, or oat dwarf virus) by sequencing [103].

### 3.4. Nucleic Acid Sequence-Based Amplification

NASBA is based on serial steps of transcription and reverse transcription processes [104]. NASBA can amplify either RNA or DNA templates, but RNA is more widespread as a target. The linear stages of RNA NASBA are: DNA oligonucleotide containing T7 promoter sequence at the 5′ end anneals with target RNA at 65 °C, AMV revertase synthetizes cDNA at 41 °C, RNAseH degrades RNA in complex cDNA–RNA, second primer binds cDNA, and AMV extends it. Finally, dsDNA containing T7 promoter is present in the reaction. The dsDNA is involved in the amplification cycle that starts from multiple RNA transcription by T7 DNA polymerase and is followed by AMV–RNAseH reaction processes with the RNA. DNA NASBA has a preliminary denaturation stage for the synthesis of cDNA from sdDNA. As the product of NASBA is RNA molecules, only labelled probe hybridization is available for detection. Most products are detected by quantification of fluorescently labelled probes or immuno-detection [105]. NASBA can be used for simultaneous detection of several pathogens [106].

NASBA is not a popular method for detecting plant pathogens. Only one research team has developed relatively rapid NASBA-based testing for the detection of Xanthomonas citri [107]. Scuderi et al. used the commercial Nuclisens basic kit (bioMérieux, Marcy l’Etoile, France) for amplification of a specific region of the pathogen (120 min at 41 °C) followed by hybridization of the amplicon on a nylon membrane. The amplified DNA was cross-linked by UV radiation. Then, hybridization procedures were performed with the Hybrimax device (Hybribio, Chaozhou, China), which provides flow-through hybridization. However, the overall process comprised prehybridization, hybridization with dioxygenin-labelled DNA oligonucleotide, washing, incubation of anti-dioxygenin antibodies, washing again, and colour developing stages. Even using the device requires 30 min for hybridization and detection. The test was able to detect 1 fg of target RNA. The rest of the NASBA-based plant pathogen tests use real-time fluorescence detection for sugarcane yellow leaf virus [108], apple scar skin viroid [109], strawberry vein banding virus [110], Arabis mosaic virus, apple stem pitting virus, potato virus Y, *Fusarium poae* and *Ralstonia solanacearum* [111], and apple stem pitting virus [112].

### 3.5. Helicase Dependent Amplification

HDA uses DNA helicase instead of the thermal denaturation of the dsDNA matrix. Original HDA utilizes uvrD helicase of *E. coli* that unwinds double stranded DNA, which are bound by single-stranded DNA-binding proteins (SSB) and allows primer binding, and extension by DNA polymerase (Klenow fragment). This HDA proceeds at 37 °C using reparation protein MutL for activation of uvrD helicase and was called mesophilic HDA (mHDA) [113]. Another modification of the HDA is based on using thermostable uvrD helicase and Bst DNA polymerase that function at 60–65 °C. This approach was called thermophilic HDA (tHDA). The proteins SSB and MutL are not necessary for tHDA [114]. These modifications make tHDA more appropriate for commercial kits. As described earlier, HDA is quite tolerant to inhibition, and it can amplify in human blood. The disadvantage of HDA is the relatively slow reaction, with a recommended duration time of 1 h [113,114]. HDA is broadly used in human pathogen diagnostics, is most similar to PCR, and can be detected in the same ways, such as through gel electrophoresis, staining, and fluorescent detection. On-site visualization methods are also used, including LFA [115,116], chip devices [117,118], and GNP aggregation [119].

There are a few tests based on HDA for the detection of plant pathogens [120,121]. The only research paper that has been devoted to visual HDA detection was designed for *Phytophthora kernoviae* [120]. Total DNA was extracted from *Rhododendron ponticum* with the innuPREP MP Basic kit. Excess of one biotinylated primer would cause asymmetric HDA. A commercial kit for HDA was utilized. Biotinylated ssDNA was added to a specific probe at the dot chip. After washing, enzymatic-dependent silver NP growth was performed with a commercial kit, and dots containing amplified DNA became grey coloured. Sensitivity of the test was 1 pg/μL pure genomic DNA. The paper was not particularly suitable for field detection because of the long duration for amplification (90 min) and visualization (60 min). The washing steps made the test even less compatible with field diagnostics. Portable heat blocks accurately keeping temperature are also acceptable for LAMP, HDA, RCA, and NASBA.

## 4. Visualization of DNA Amplification Products

### 4.1. Coloration for Visual Detection and Fluorescence for UV Lamp Detection

The easiest method for visual equipment-free or low-equipment detection after amplification is colouration. There are methods for colour development for DNA-containing solutions. One method is the direct binding of a dye with DNA molecules. dsDNA molecules can interact with dyes in three different ways: The dye can intercalate between strands of the DNA, it can bind with a major groove of the DNA, and it can bind with a minor DNA groove. The most common mechanism of detection is intercalation corresponding with ethidium bromide, SYBR Green I (SGI), propidium iodide, and SYTO-16, among others. The key property of these dyes is an increase of fluorescent intensity upon dsDNA binding [122]. SGI is the most popular dye for post-reaction and real-time detection of dsDNA. Maximum absorption of SGI is 497 nm (blue), and extinction peak wavelength is 520 nm (green). It is relatively cheap, has low toxicity, and has up to 2–3 orders of difference in fluorescence between dsDNA- and ssDNA-bound forms [123]. SGI has some drawbacks, however. It has moderate stability [124], and there can be inhibition of some amplification (LAMP, PCR) at the SGI range of optimal fluorescence [125,126]. Additionally, SGI interacts with ssDNA primers with lower affinity [123]. This can complicate the interpretation of the results by comparing negative samples and samples with negligible amplification of the target. Not all amplification methods produce dsDNA; for example, RCA generates ssDNA molecules that can be specifically detected by cationic dye, such as QATPE [127].

Another type of colourization is the use of dyes for by-products from the amplification process. These methods are amplification-dependent and rely on different compounds. Some use additional enzymes (e.g., alkali phosphatase or horseradish peroxidase) or specific external DNA/RNA to form noncanonical DNA structures in amplified products [128]. Specific approaches for colorimetric visual detection have been designed for RPA [129,130], RCA [131], NASBA [132], and, most popular, LAMP. One of the most sensitive and easy-to-use dyes for LAMP detection is hydroxy naphthol blue (HNB). During LAMP, pyrophosphate is produced as a by-product, which reacts with magnesium to form the precipitate magnesium pyrophosphate. The pH of the LAMP mixture changes with decreasing magnesium concentration which changes HNB from violet to sky blue (absorbance at 650 nm) [133]. Magnesium pyrophosphate precipitation can also be detected during the amplification reaction, per sec [134]. Other pH-sensitive dyes such as phenol red, cresol red and neutral red have also been designed for visual detection of LAMP products. The major advantage of this approach is that it provides an easy visual detection as positive and negative samples have different colour spectra [135].

Portable equipment for real-time amplification with fluorescence detection is used both in and out of the laboratory [136]. Portable equipment for on-site amplification and real-time fluorescence detection is available for purchase and applied for on-site pathogen detection. Different types of devices are used for RPA [137] and LAMP [138,139] for plant pathogens, providing more reproducible diagnostics. The quantitative in-field results obtained would allow for the estimation of disease severity on site.

#### 4.1.1. Colorimetric Detection of RPA Amplicons

SYBR Green detection using UV light, a colorimetric assay and a portable device was designed to identify *Bursaphelenchus xylophilus* extracted from pinewood chips [137]. A 129 bp product synthetized from ribosomal intergenic spacer for 20 min was visualized using SYBR Green. Pure genomic DNA was detected from 1.6 fg. The research shows a 10-fold higher sensitivity for colorimetric RPA than real-time LAMP for spiked samples. According to Cha et al. [137], measurements of adsorption was more appropriate for detection as they visually could not differentiate colour changes. However, the portable device used can cause some restrictions for in-field applications. Therefore, UV visualization may be better suited for in-field diagnostics, but it can also lead to false positive results.

#### 4.1.2. Colorimetric Detection of LAMP Amplicons

The simplest detection of LAMP products is through colorimetric assay. During the design of LAMP analysis, SGI or other colorizations are used for additional detection, besides gel electrophoresis and quantitative analysis. In such cases, visual detection is a by-product of the assay and can be used easily for in-field diagnostics. We will consider each of these methods separately.

Many articles describe using SGI with either UV light or the naked eye for detection. Different groups of plant pathogens have been detected by LAMP with SGI, including bacteria [140,141,142,143], oomycetes [144], fungi [145,146,147,148,149], and viroids [150,151]. Most pathogens detected by SYBR are viruses [152]. For RNA viruses, LAMP is combined with reverse transcription. Some LAMP tests propose SGI detection without UV light, making the tests cheaper and easier [10,153,154,155,156].

The most observable colorimetric method of detection is those that involve pH changes. A developing yellow colour in a positive LAMP reaction is easily distinguishable from a pink negative sample [157,158,159,160]. The opposite colorization is also available [161,162,163]. There are commercial premixes containing pH indicators that can provide detection without opening the tube after the LAMP mix to prevent possible contamination. HNB dye can change from purple to sky blue during reactions. HNB detection for viruses [164,165,166,167,168], bacteria [169,170], and fungi [171,172,173,174] has also been developed. There is only a prototype of direct detection of plant pathogen via magnesium pyrophosphate precipitation [175].

#### 4.1.3. Colorimetric Detection of RCA Amplicon

SBI has also been used with RCA and tests for plant pathogens have been developed. The simplest means of visualization was proposed for PLP–RCA detection of four species of Neofabraea fungi (N. perennans, N. kienholzii, N. vagabunda, and N. malicorticis) [176]. The test focuses on discrimination of the four species, rather than sensitive detection with 107 copies of the target gene (elongation factor 1a) in the reaction. The fungal DNA is extracted from apple roots using the MagPure Fungal DNA TL Kit. SGI is used for visualization of the DNA product. Duration of the assay was 110–140 min.

An RCA–PLP test for rapid differential diagnosis of the fungal pathogens Fusarium graminearum species complex, F. oxysporum, F. incarnatum-equiseti, and F. tricinctum has been developed [177]. Fungal DNA was extracted from plant tissues using the CTAB method and genes for elongation factor 1a were chosen as targets. The products of amplification were visualized using SGI in UV light and verified using gel electrophoresis.

One interesting study used Cas9 triggered strand displacement amplification followed by RCA [178]. Firstly, the amplification stage begins when the Cas9-sgRNA complex recognizes the target model DNA of Phytophthora infestans and makes a cleavage in one strand. Next, Bst polymerase with strand displacement activity synthetizes ssDNA. The ssDNA copies become a matrix for PLP–RCA. The long ssDNA product can bind with GNP conjugated with short complementary DNA oligos, causing aggregation of DNA, and a change in colour. Sensitivity to the model fragment with this assay is 0.2 pM. Extremely high specificity of Cas9 recognition could make this approach a prospect for laboratory diagnostics. However, the concept of the test suggests too much enzymatic reaction, which would limit its applicability for field use.

### 4.2. Nanoparticle Aggregation

Colour change during the transition of individual nanoparticles (NPs) in solution to their aggregated state is a fairly rapid and effective tool for detecting compounds including DNA that cause aggregation [179]. The NP aggregation-based method is visualized with colour reactions as well as the reactions described in Section 4.1. However, the use of NPs provides higher sensitivity and more flexible configuration for detection [179]. The simplest visual detection of DNA is based on the aggregation of GNPs. DNA binding with GNPs can be achieved in covalent and noncovalent ways. Noncovalent binding efficiency depends on GNP size and DNA composition, type, and length. The binding type is more favourable for ssDNA shorter than 100 nt. Weak interaction of dsDNA has been established only for a particular size of GNP and dsDNA length [180]. Some ssDNA coupled with GNPs can prevent salt-induced aggregation of GNPs. The presence of complementary ssDNA in solution abrogates this effect. Colloidal solutions of GNPs are coloured red, but aggregated GNPs become purple. Colour-change in the presence of target ssDNA by functionalized GNPs is a base for visual detection [180,181]. Different variations of covalent and noncovalent DNA bound to GNP are used for the detection of pathogens after DNA amplification followed by the hybridization of ssDNA or melted dsDNA [182,183]. This approach has found an application in the detection of plant pathogens in combination with two amplification methods, RPA and PCA.

#### 4.2.1. RPA Amplicon Detection with Nanoparticle Aggregation

An interesting approach was proposed by Wang et al. for the detection of tomato yellow leaf curl virus [184]. A thiol-labelled DNA probe was added to an RPA mix after 10 min at 33 °C. The amplicons then denatured at 95 °C, and hybridization with the probe took place for 5 min. Two chemical compounds (H_2_NOH and HAuCl_4_) were added to the mixture to grow GNPs. Conjugation of GNP–DNA happened during the GNP synthesis. In the case of the dsDNA conjugate, GNP aggregated and had a blue colour, instead of pink. Gradual changing of the coloration began from initial concentration of one copy per µL, but naked-eye detection was at 106 initial copies. Reference qPCR was able to detect one copy per µL. The method is quite sophisticated for application in the field. Special compounds are required for it, and therefore, the method will be difficult to scale in practice.

#### 4.2.2. RCA Amplicon Detection with Nanoparticle Aggregation

A prototype test has been developed from RCA to detect tomato leaf curl New Delhi virus based on the sandwich hybridization of amplified genes of plant viruses with ssDNA probes linked with glass slides and GNP–ssDNA conjugate [185]. After two serial hybridization washing cycles, aggregated GNPs were detected by a portable scanner. The test demonstrates high sensitivity (100 zM–100 aM of pure DNA fragment), but it is too complicated for an on-site detection assay and has a long duration (2 h).

### 4.3. Lateral Flow Assays

**Figure 4:**
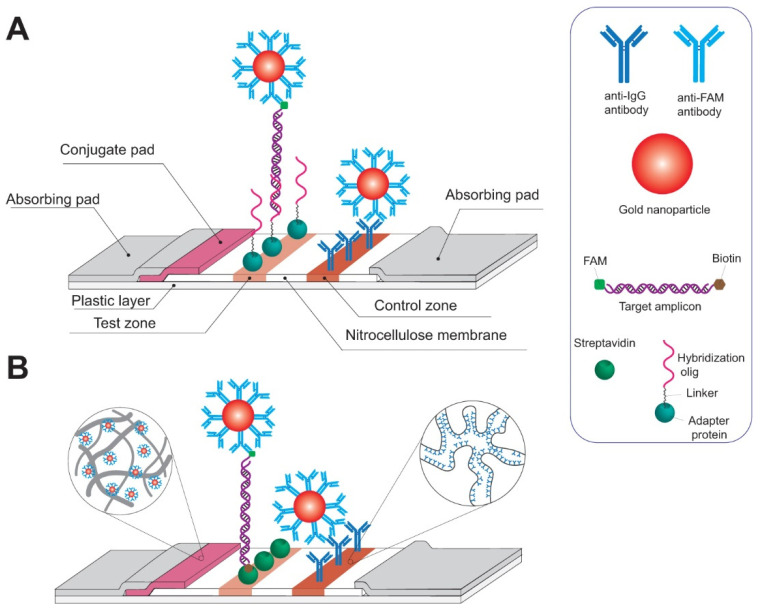
Schematic of a lateral flow assay for detection and visualization of DNA amplification products: direct detection of DNA sequences (**A**) and nucleic acid lateral flow immunoassay (**B**).

Lateral flow assays (LFAs) are a rapid, simple, and cheap way to detect amplicons. Since the 1980s, LFAs have been used extensively in diagnostics for clinical and veterinary medicine, agriculture, and food chemistry, to monitor environment pollution, and primarily as an immunoassay tool [186,187,188,189,190]. The method is based on the separation of molecules flowing through test strip membranes followed by the detection of target molecules in a test zone using coloured labels (Figure 4). Due to the pore structure of the membrane, large macro compounds, cells and fragments in the sample, are separated from smaller compounds via diffusion [189]. This is similar to NP-based methods as the detection label is most often nanoparticles. Different NPs can be used for conjugation, such as colloidal gold, coloured latex, fluorescent particles (e.g., quantum dots), and carbon particles [191,192]. However, in contrast to the NP aggregation methods described earlier, in LFA, specific affinity recognition of the target occurs owing to receptors (most often antibodies) being immobilized on the NP surface. NPs are concentrated in the binding (test and control) zones as a result of the formation of specific complexes that lead to colorization in these zones. The typical time taken for LFA is 10–20 min, which makes it a highly rapid and convenient detection tool for in-field diagnostics.

Two approaches are used for the detection of nucleic acid targets with LFA: direct detection of DNA sequences and nucleic acid lateral flow immunoassay (NALFIA) (Figure 4). The first method consists of specific hybridization of single-stranded nucleic acid targets with complementary nucleotide fragments immobilized in the test zone [193,194,195,196]. The second is more common and is based on the labelling of DNA molecules with low-molecular-weight labels, including biotin, fluorescein, and digoxygenin. The most common method is an amplification of target DNA or RNA with 5′ labelled primers: one primer is biotinylated, while another is labelled with FAM. Following this, the analysed sample, after amplification of target RNA/DNA, is applied to LFA to provide formation complex streptavidin—biotin-dsDNA-FAM—anti-FAM antibodies conjugated with NPs in the test zone. Furthermore, a combination of the hybridization type and NALFIA has been proposed in some papers [197,198]. Target DNA is amplified with only a labelled primer, then high-temperature (above 65 °C) hybridization with labelled oligonucleotide probe is performed.

Unlike all other methods for the detection and visualization of amplicons, LFT strips are commercially available for detecting biotin-/FAM-labelled amplicons, including those by Millenia (Gießen, Germany), BioUSTAR (Hangzhou, China), Agdia (Elkhart, IN, USA), and Abingdon Health’s PCRD nucleic acid detector (York, UK). However, to improve some applications, components of LFT strips can be modified and optimized for signal increase in the test zone [87,199]. The second important advantage of test strips is the availability of already developed portable devices to register colorization in the test zone. Portable devices are highly convenient for recording results for in-field diagnostics. Upon using the portable devices, such labels as fluorescent dye or quantum dots can decrease detection limits [200,201].

Analysis of the literature shows that a combination of isothermal amplification and LFT strips are the most popular and widely used methods for in-field detection of plant pathogens. The most commonly used amplification methods with test-strips are RPA and LAMP, and there is some use with HDA and NASBA. To date, there are no LFA-based detections for plant pathogens using RCA. However, RCA–LFA has been developed for other organisms. To detect the algae *Karenia mikimotoi* in the environment, hyperbranched PLP–RCA has been performed with a biotin-labelled primer. Then, an FITC-labelled probe was hybridized with the ssDNA product and applied to LFA [202].

#### 4.3.1. RPA-Based Tests

RPA of plant pathogens has been described in approximately 50 original papers. However, authors of only 16 of these used LFA (Table 1), one used SYBR Green, and another used GNP aggregation for visualization. Of 20 reported pathogenic species comprising bacteria, viruses, viroids, fungi, and oomycetes, 11 were rated as having the most significance for plant pathogens [203,204,205,206,207]. We collected different aspects (e.g., detected target species, gene, host organism, sensitivity, time of detection, reference method and its sensitivity, method for RNA/DNA extraction, time of extraction) of the RPA tests and have presented these in Table 1. Analysis of the table can be useful for developing new tests in the future.

First, all these tests were performed with the following commercial RPA kits: TwistDx nfo [27,84,208,209,210,211], TwistDx Basic [212,213,214,215], TwistDx Exo [51], and Amplifier (Agdia) [27,92,216,217,218]. The main criterion for the selection of a gene as a target for RPA was maximal specificity for the species of interest. As stated earlier, RPA has low sensitivity to nucleotide variations. In the case of virus pathogens, the researchers chose a gene sequence within the coat protein for all viruses. Viral coat proteins are quite conserved [219]; however, there is enough variation to differentiate species [220]. In addition, the RNA/DNA of coat proteins is fairly abundant compared to other viral regions [221]. In case of bacteria or eukaryotic pathogens, known species-specific genomic marker sequences were chosen; for example, ribosomal gene [217] or ribosomal intergenic sequence [137,222]. The reviewed papers describe using primers with optimal length and amplicon size in the range of 100–300 bp.

Most researchers used a canonical THF probe for Nfo cleavage and detection. One test utilized a probe containing 1,2-deoxyribose instead of THF, which would lower the price of probe synthesis [27]. In another study [51], the TwistDx exo kit was used. The kit is similar to the Nfo kit, using the ExoIII nuclease instead of the Nfo one. Other papers have proposed different approaches [212,215]. The authors used TwistDx basic kit without cleavage and no complex DNA probe. RPA was performed with FAM- and biotin-labelled primers that were picked after screening. This simplification has previously been applied in other RPA papers [212,213,214,223]. Of course, an additional probe in RPA should enhance specificity and decrease background on the test dipstick. However, it is possible to create simpler RPA tests with optimized labelled primers that do not form stable cross-dimers in LFA. Cross reactivity of the chosen primers with relative pathogens was tested in most of the papers. The tests were validated with infected plants and spiked samples. In the case of SYBR visualization, primers were designed to avoid false positive staining of the reactions [137].

Most of the authors used commercial kits to extract DNA or RNA from samples. These provide similar conditions for DNA extraction through relatively rapid processes. Extractions from crude plant homogenate were also performed in most of the studies. Crude extraction enables significantly accelerated and cheaper analysis [26,216]. The amount of extracted nucleic acid can be significantly decreased within crude extraction, so some researchers used lateral flow dipsticks/discs for high-capacity, rapid, and specific extraction of nucleic acids [83]. Compared with PCR (approximately 1 h after extraction of nucleic acids), these tests gave faster results, with most being done within 1 h. However, generally, they are 10–100 times less sensitive than qPCR although some tests demonstrated similar sensitivity [82,210,212,214,216] or higher [208,218,224,225] than either qPCR or PCR. LFA dipsticks can detect amplified products less-than-routine gel-staining amplicons after PCR or RPA. Most plant RPA tests use GNP–anti-FAM conjugate LFA test strips. Some tests utilize anti-digoxygenin conjugate with carbon NPs in the test zone [211]. The commercial LFA kits were purchased from Millenia [51,208,209,210], PCRD nucleic acid detector [84,211,222], BioUSTAR [26], and Agdia [27,92,216,217,218] or assembled and optimized from different membranes (homemade test strips) [212,213,214]. RPA contains a large amount of DTT that induces aggregation of some GNP conjugates, such as streptavidin–GNP [87]. PCRD strips can avoid this restriction because carbon NPs are used for conjugates.

Thus, a comparison (Table 1) of RPA-based tests for plant pathogens with visual detection shows that, despite many visualization approaches for RPA, LFA is mostly used for detection for plant pathogens, SYBR Green visualization is less commonly used for pathogens, NP-based tests are too complicated for field diagnostics, and there is no microfluidic-based chip detection for plant pathogens, despite many approaches for RPA to detect other pathogens [226], and THF-probe was utilized in most of them. Overall, on average the time of analysis was within 2 h. Analysis can be accelerated by disc extraction of DNA from crude homogenate. Summarizing the considered RPA–LFA tests for plant pathogen detection, we can conclude that RPA–LFA is 10 times less sensitive than quantitative PCR. Therefore, RPA–LFA could be applied for in-field diagnostics and provide the highest sensitivity among the field methods.

**Table 1:** Combination of RPA–LFA methods for plant pathogen detection.

Detected Target Specie, Gene	Host Organism	Detection Limit	Time of Detection, Min	Reference Method and Its Detection Limit	Method of RNA/DNA Extraction	Time of Extraction, Min	Ref
Potato virus X * (gp5 gene, 147 bp)	Potato leaves	0.14 pg virus per gram of plant leaf (spiked samples)	30	RT–qPCR: 0.14 pg virus per gram of plant leaf	Syntol kit	30	[212]
Potato spindle tuber viroid *	Potato tuber	10^6^ copies of in vitro transcribed PSTV RNA, up to 10^7^ dilution of infected plant	30	RT–qPCR: up to 10^7^ dilution of infected plant	Syntol kit	30	[214]
Tomato spotted wilt virus ** (coat protein)	Pepper leaves	10 fg/μL of transcribed TSWV RNA	15	RT–PCR: 10 fg/mcL of transcribed TSWV RNA	TRIzol (Thermo Fisher Scientific) extraction	40	[210]
Citrus tristeza virus * (coat protein)	*Citrus aurantiifolia*, *C. sinensis*, *C. reticulata*	For transcribed in vitro RNA: 141 fg (3.77 × 10^5^ copies) For native RNA: 6.288 × 10^6^ copies	25	RT–qPCR: for transcribed in vitro RNA: 141 fg (3.77 × 10^3^ copies)	RNeasy Plant mini kit (Qiagen)	<20	[211]
Milk vetch dwarf virus ** (coat protein)	Cowpea	Plasmid with cloned fragment of MDV spiked with crude extract: 10 copies/μL	40	RT–qPCR: plasmid with cloned fragment of MDV spiked with crude extract: 10 copies/mcL	E.Z.N.A.^®^ Plant DNA Kit (Omega Bio-tek)/crude extraction	Approx. 40/<5	[215]
Rice black-streaked dwarf virus * (P10 gene NC_003733.1, approx. 200 bp)	Rice leaves	10-fold dilution of cDNA (Milenia test-strips)	25	RT–qPCR: 10^3^ dilution of cDNA	RNAiso Plus Kit (TAKARA)	60	[227]
Hop stunt viroid 140 bp (101 labelled)	Leaves of hops, cucumbers, plums, grapes, and citrus	2 × 10^9^ copies transcript in crude extract (Agdia test-strips)	40–50	RT–PCR: 2 × 10^4^ copies transcript in water	RNeasy Plant Mini Kit (QIAGEN), also crude homogenization	<20/<5	[27]
Tomato chlorotic dwarf viroid * 228 bp (131 labelled)	Tomato seeds, leaves	1 pg transcript, 1:25 dilution of leaf extract, 1:10 dilution of seed extract (Agdia test-strips)	35	RT–PCR: same	AmplifyRP^®^ Acceler8^™^, crude extract	<5	[216]
Plum pox virus * (coat protein, 147 bp)	*Prunus* leaves	1 fg transcribed RNA, 1:10000 crude extract (Agdia test-strips, TNF probe)	35	Real-time RPA: 16 fg transcript. Real-time RT–PCR: 10 fg RNA	SurePrep^™^ Plant/FungiTotal RNA Purification Kit (Thermo Fisher Scientific)/crude plant extract	30	[218]
Little cherry virus 2 ** (coat protein, 134–295 bp)	Cherry budwood or leaf	Crude extract 1:100, 0.1 ng of pure total RNA (Agdia test-strips, nfo probe)	25	RT–PCR: crude extract 1:10K	RNeasy Plant Mini Kit/crude extract	<20/<5	[92]
*Dickeya solani* * (SOL-C genomic region)	Potato tubers	14000 CFU per gram of plant leaf (spiked samples)	30	qPCR	Syntol kit	30	[213]
*Dickeya solani*, *D. chrysantemi*, *D. dianthicola*, *D. dadantii*, *D. paradisiaca*, *D. zeae*–overall 34 strains (mglA/mglC genomic region)	Potato tubers, sweet potato tubers, taro corms	1 CFU of *D. dianthicola* (purified bacteria or spiked samples) Real samples with other *Dickeya* species—pos/neg	35	PCR followed by sequencing (qualitative confirmation)	Wizard Genomic DNA Purification kit/crude extraction	120 min	[51]
Genus *Clavibacter and C. nebraskensis in particular* *	Corn leaves	3000 copies of genomic *Clavibacter* and 30 copies of genomic *C. nebraskensis*. 3000 copies of genomic *Clavibacter* and 300 copies of genomic *C.nebraskensis* in spiked samples	35–40	PCR for qualitative confirmation	Crude extract in TE buffer	5–12	[84]
*Phytophthora hibernalis* ** Ypt-1 gene, approx. 200 bp	Orange fruit crop	0.2 ng (extracted from *P. hibernalis*), pos/neg for artificially inoculated plant (milenia test strips)	25	PCR: 2 ng extracted from *P. hibernalis*	DNAsecure Plant Kit (Tiangen Biotech)	20	[224]
*Phytophthora sojae* ** Ypt-1 gene, 217 bp	Soy seeds	0.01 ng genomic DNA (milenia test strips)	25	LAMP: 0.1 ng genomic DNA [216] PCR: 1 ng genomic DNA [217]	DNAsecure Plant Kit (TIANGEN)/FastDNA SPIN Kit for Soil/NaOH lysis method [40]	20/30/<10	[225]
*Phytophthora capsici* Ypt-1 gene	Potato leaves	10 pg genomic DNA, Pos/neg for infected plant (Milenia test strips)	25/15	LAMP: 100 pg genomic DNA, real-time qPCR: 100 fg	HP Fungal DNA Kit (Omega Bio-Tek)/Cellulose dipstick capture of DNA [49]	Approx. 25/<5	[83]
*Phytophthora infestans* ** Ypt-1gene	Potato leaves	500 fg of genomic DNA (approx. 2 genome copies) from the bacterial isolates Pos/neg for infected plants	25–35	Conventional PCR: 5 pg	PEG lysis	5	[208]
*Phytophthora cactorum* ** Ypt-1 gene	Strawberry leaves	100 fg of genomic DNA	35	Conventional PCR: 1 pg	DNAsecure Plant Kit (Tiangen)/PEG lysis	20	[209]
*Candidatus Liberibacter asiaticus* ** 16S rRNA gene, 170 bp	Sweet orange fruit, acid lime leaves	<1 pg total DNA (with PCRD nucleic acid Detector and Agdia)	30	Real-time PCR: 10–100 fg of total DNA	DNeasy Plant mini kit/crude extraction	20/<5	[222]
*Pectobacterium*. *Carotovorum* * subsp. *carotovorum;* *P. carotovorum* subsp. *odoriferum;* *P. carotovorum* subsp. *brasiliensis;* *P. atrosepticum;* *P. parmentieri*	Tomato fruit, potato tuber	10 fg DNA for either purified bacterial DNA or purified spiked samples (Milenia test-strips)	35	None	Wizard Genomic DNA Purification K/Plats with inoculated bacteria were homogenized in TE buffer	120	[228]
*Gaeumannomyces avenae* **	Non-identified roots	100 pg genomic DNA	40	LAMP: 1000 fg	Crude extract	<10	[217]
*Ophiosphaerella korrae* **		100fg (Agdia test strips)		1 fg			
*Magnaporthiopsis poae* **		1 fg		100 fg			
*Candidatus Phytoplasma oryzae* ** imp gene KU820961	Napier grass	10–100 copies target DNA in water, pos/neg for plant extract (BioUSTAR test strips)	25	Real-time RPA: 1–10 copies of target DNA/PCR for plant extract	CTAB method/homemade homogenization method	Data not provided	[26]

* test system was validated with “artificially” infected plant samples (infection by inoculation or selection in an experimental greenhouse. ** test system was validated by plant samples collected from wild or commercial field.

#### 4.3.2. LAMP-Based Tests

LFA detection of LAMP uses labelled primers in different combinations (e.g., inner primers [229], loop primers [230], and inner and loop primers [231]). Another method of detection is hybridization of LAMP products with hapten-labelled probes after amplification [198]. A method of co-elongation including biotin- and FITC-labelled nucleotide has been proposed [232].

The total number of LAMP-based tests reported in papers stands at more than 100. Although LAMP is the most popular isothermal amplification [98], only a few original papers reported developing LAMP–LFA-based tests for 11 plant pathogenic species (all are presented in Table 2). We analysed these papers to find common approaches for LAMP–LFA. High-variable sites of genomes were selected for primer annealing. Like RPA tests, coat protein sequences were used for LAMP detection of some viruses [233]. Additionally, intergenic ribosomal spacer sequences were picked for some pathogens [230,234,235]. Most of the LAMP–LFA was developed as a point-of care test without sensitivity estimation and comparison with PCR. The LAMP–LFA tests that were verified by PCR demonstrated similar sensitivity to the reference method.

Lateral flow strips from different manufacturers—Millenia [197,234,235], Foresite Diagnostics [230,233,236], Biohelix [229], and AMODIA Bioservice [237]—were implemented for the detection of labelled amplicons. Additionally, homemade dipsticks utilizing NP–streptavidin conjugate were used [237]. Different approaches were designed to obtain labelled products, including FIP/BIP-labelled primers [229,236], FIP-/loop-labelled primers [237], loop-labelled primers [230,233], and biotin-labelled probes for hybridization of FITC-labelled amplicon [197,234,235]. The overall analysis comprised extraction of DNA, amplification, optional hybridization, and LFA. LAMP required 30 min at least, LFA required 5 min, and hybridization required 5 min, although the time for extraction could vary significantly. An interesting solution was proposed by Tomlinson [230], whereby a lateral flow dipstick extraction of DNA from plant samples took from 8–10 min. The tests of cassava brown streak virus and Ugandan cassava brown streak virus were developed as multi-pathogen detection lateral flow dipsticks [233]. Labelling with TexasRed and digoxygenin for one target and FITC–biotin for another allows the use of lateral flow test strips with two test zones.

A comparison of LAMP-based tests for plant pathogens with visual detection showed that LAMP appears to be quite popular for field diagnostics. However, a few LAMP–LFA tests have been designed, and most of these use the simplest means of detection, namely visualization by SGI or another dye. The sensitivity of this approach is similar to PCR or canonical LAMP.

**Table 2:** Combination of LAMP–LFA methods for plant pathogen detection.

Detected Target	Host Organism	Detection Limit	Time of LAMP-LFA, Min	Reference Method	DNA Extraction	Time of Extraction	Ref
Cassava brown streak virus and Ugandan cassava brown streak virus ** (Coat protein)	Tobacco leaves	Pos/neg	55	Realtime PCR, LAMP, PCR	CTAB method	>8 h	[233]
Tobacco rattle virus and potato virus X **	Potato	Positive/negative	55	RT-qLAMP–15 pg, RT-qPCR–15 pg, Pos/Neg tests: RT-PCR, RPA (TwistAmp Basic, AmplifyRP Acceler8 Discovery Kit), IsoAmp II Universal tHDA Kit (NEB), CRISDA	PureLink Plant RNA Reagent protocol/Modified PureLink Plant RNA Reagent protocol/Potato DNA/RNA rapid extraction set/InCus based on Monarch Total RNA Miniprep Kit (NEB)/crude extract	>60 min	[238]
*Clavibacter michiganensis* subsp. *sepedonicus* 16 S rDNA intergenic spacer region AF001266.1	Potato tuber	Pos/neg test (validation of LAMP)	70	LAMP	SureFood PREP Advanced Kit (CONGEN)	65 min	[235]
*Leifsonia xyli* subsp. *Xyli* ** ISLxx5 transposase gene NC_006087.1	Sugarcane xylem sap and leaves	Pos/neg test, 1:5 diluted infected plant extract	40	LAMP	Homemade method	<20 min	[236]
*Candidatus Liberibacter asiaticus* **	Sweet orange leaves, *Diaphorina* *Citri* fly	10 pg purified DNA from infected plant	45	Real-time PCR: same	Wizard^®^ Genomic DNA purification Kit (Promega)	Approx. 120 min	[197]
*Xanthomonas citri* ** Scheme of the complex looks like a padlock *PthA4* gene XACb0065	Lime leaves	1 fg pure DNA, 5.2 CFU pure culture per reaction, 18.7 CFU from infected tissue per reaction	>30	Conventional LAMP: same	Wizard^®^ Genomic DNA purification Kit (Promega)	Approx. 120 min	[229]
*Aspergillus fumigatus* ** anxC4 gene	No plant objects were tested	100 fg of genomic DNA	52	Culture method and PCR: 100% correlation with the LAMP	QIAamp DNA Mini Kit	20	[237]
Phytophthora ramorum, P. kernoviae ** ITS 1 region of the nuclear ribosomal (nr)RNA gene	Rhododendron leaves	17 pg purified genomic DNA (from fungi culture)	>60	LAMP: 10 pg [220]	NucleoSpin Plant kit (Machery-Nagel)/homemade method based on lateral flow dipstick	35 min/<10 min	[230]
*Aspergillus sp.* ** aflR gene	Different herbal samples	10 copies of the gene in buffer	30 min	LAMP: the same PCR: 100 copies of the gene	The Wizard^®^ Magnetic DNA Purification System for Food	<50 min	[239]

* test system was validated with “artificially” infected plant samples (infection by inoculation or selection in an experimental greenhouse. ** test system was validated by plant samples collected from wild or commercial field.

#### 4.3.3. NASBA-Based Tests

NASBA is mostly used to detect human viruses (e.g., HIV) [105] and is unpopular for the detection of plant viruses. Although there are a few papers on the topic [106,109,240,241,242,243], none of the research can be applied to rapid field diagnostics. These detections comprise gel electrophoresis, fluorescent quantification, and chemical linking of NASBA product for flow hybridization. LFA detection of NASBA products has been developed for other pathogens, including the dengue virus [244] and HIV [245]. LFA has not been proposed for plant pathogen detection. Indeed, NASBA has several drawbacks that make it unsuitable for field applications, namely that it is expensive and has thermo labile enzymes and a short-amplified region (up to 250 nt). In addition, a revertase and T7 RNA polymerase should be added separately, which increases contamination risk [105].

### 4.4. Lab-on-a-Chip

Chip tools are not specific DNA detection methods but paper-based or microfluidic devices for a combination of amplification reaction and detection. There are many DNA detection types that are compatible with isothermal amplifications, such as fluorescence, colorimetry, and NP detection. The use of lab-on-a-chip is accurate, rapid, and portable, and it can reliably detect pathogens [138,246]. Chips are compact devices, but some require the use of unique and sophisticated equipment for their manufacture or signal interpretation [247,248,249]. Here, we consider portable and autonomous chip biosensors that can be used on-site and without lab access. Only a few tests that satisfy these requirements were proposed that were based on a combination of lab-on-a-chip with LAMP. Thus, various microfluidic devices perform LAMP and detect its products [250,251]. Different sources of DNA and RNA can be used for lab-on-a-chip devices. In this review we have focused on paper-based lab-on-a-chip approaches that utilize simple, complex, and technological detection. Not only are pathogens detected using this approach but also genetically modified organisms [252]. Two lab-on-a-chip assays were proposed for the detection of plant pathogens. A microfluidic device for the detection of viruses from orchid leaves was designed by Chang [253]. However, this test requires complex and unique equipment that supplies temperature regulation, has a vacuum pump, and detects optical density in a chamber of the chip. In fact, the test cannot be used in field diagnostics.

The second test for diagnosing seven species of *Aspergillus* was based on a paper chip device that is easier to manufacture and use [239]. The chip consists of two parts for reaction and detection. Each of the zones contains a paper disc for absorption of the compound. LAMP proceeds through paper in the reaction zone, and then a solution with DNA product is squeezed into another zone through capillaries; GNP–ssDNA conjugate in low salt had previously been injected into that zone. If target LAMP products are present in the zone, they form a complex with the GNP–ssDNA during hybridization and prevent salt-induced GNP aggregation. The blue colour of the GNP does not change in this way, but in the case of aggregation, it loses its colour. The conjugate after hybridization is squeezed toward another paper disc for better visualization.

## 5. Conclusions

On-site detection of a plant pathogen requires user-friendly and short-term stages of plant tissue processing, nucleic acid extraction, amplification, and detection. To date, existing portable and user-friendly equipment, kits, and protocols allow for performing an on-site preparation for plant DNA/RNA extraction. Features of target plant tissue should be taken into consideration and proper homogenization methods should be chosen. Processing of soft tissues or exudate can be performed within 5 min by crude equipment-free homogenization. For fine homogenization and use of inhibition-tolerant amplifications for a pathogen can be in crude extract, omitting the DNA purification stage. Isothermal amplifications are most appropriate for this because they contain features for on-site diagnostics

**Figure 5:**
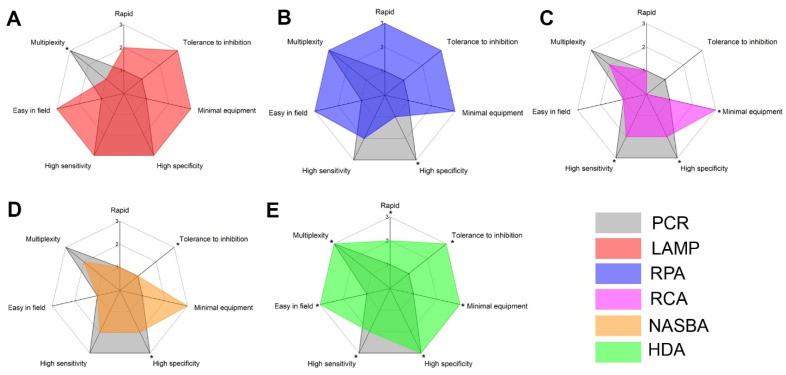
Multiparametric comparison of isothermal amplification methods for plant pathogen detection. PCR as “gold standard” of diagnostic is compared with: (**A**). loop-mediated amplification, (**B**). RPA, (**C**). rolling circle amplification, (**D**). nucleic acid sequence-based amplification, (**E**). helicase-dependent amplification. The parameters are ranged by analysis of publications. Numbers represent arbitrary pronouncing of the corresponding parameters based on corresponding PCR parameters: 0. No data, 1. Weak, 2. Intermediate, 3. High (see additional specifications in Supplementary Information, Section S2). Axes of parameters that were evaluated by non-plant data are marked by-*.

To sum up the findings from different approaches to on-site detection of plant pathogens, we summarized the amplification of pathogen DNA/RNA and visual detection of amplicons as a comparison diagram (Figure 5) and table. The aforementioned isothermal amplification methods were compared with PCR as a “gold standard”. The table comprises the main features of an ideal field-deployable analysis, namely duration and sensitivity, among others (Table 3). Each approach has advantages and disadvantages for field applications; however, none of the reviewed tests aligned with all the proposed requirements.

Obviously, isothermal amplification methods are more suited for in-field applications. However, each isothermal method has features that can restrict its use. As the most popular isothermal amplification, LAMP has widespread use in the detection of plant pathogens. Although it is cheap and easy to perform, its specificity and sensitivity are nontolerant to temperature fluctuations and the primers sets can be difficult to design. In addition, false-positive reactions due to primer cross-dimer formations are known to occur. RPA is the fastest amplification method and efficient at 35–42 °C. It is tolerant to temperature fluctuation, but it cannot discriminate up to nine mismatches in both primers which can decrease the specificity of the assay. Additionally, RPA is relatively expensive because it is produced by only one manufacturer. The other isothermal amplification methods are less popular for testing plant pathogens. RCA is less suitable for the detection of some plant viruses, considering the low percentage of plant viruses that contain DNA. Other targets require ligation and nuclease cleavage that reduce RCA’s application for field diagnostics. NASBA requires expensive enzymes that must be added separately. In addition, it is not a real isothermal method and requires a high-temperature denaturation stage. HDA is quite slow, but it can be accelerated for some means of detection. LAMP and RPA require user-friendly equipment as a block heater and can be performed in low equipment points or on-site conditions.

Visualization detection methods can also be assessed using the criteria. Although each of these detection methods can be used with each amplification method, there are preferences for some amplification methods in the case of plant pathogen detection. SYBR Green fluorescence and coloration detection are popular with LAMP. They provide rapid detection but can give “smoothed” signals. LFA is mostly coupled with RPA. Use of probes increases the specificity of RPA, but this requires complex inner modification that makes synthesis more expensive. However, terminal labelling of amplicons by modified primers is also available, which makes RPA–LFA simpler and cheaper. Occupying a niche is a simple device for amplification and detection that combines these approaches in one preliminary optimization (e.g., lyophilized amplification mix with primers) [254]. The devices are most applicable for field practice. Another means of on-site visualization is via commercially available portable devices that can measure fluorescence and detect LAMP or RPA in real time. However, the tools are a more expensive approach compared to LFA.

In summary, we recommend using LFA for rapid and sensitive detection. The dipstick can be kept for some time. Additionally, LFA contains intrinsic control of detection and can be manufactured for multitarget detection. Regarding amplification methods, we recommend RPA as the most aligned for LFA detection, and this has been verified by many studies. It can give the quickest results in combination with disc DNA extraction and LFA detection. LAMP is a good substitution for RPA in case of restricted budget for diagnostics. In the end, we wondered about the absence of an HDA–LFA test for plant pathogens. We suppose this combination could provide relatively rapid and cheap detection.

## Figures and Tables

**Table 3:** Comparison of visual detection methods of amplicons for plant pathogen detection.

Detection	Rapid	Minimal Equipment	High Specificity	High Sensitivity	Pronounced and Constant Signal	Easy to Perform in Field
SYBR Green	+	+/−	+/−	+/−	−	+
Coloration	+	+	−	−	−	+
GNP	+/−	+	−	−	+/−	+
LFA	+	+	+/−	+	+	+
Lab-on-chip	+	+/−	+	+	+	+

## Supplementary Materials

The following are available online at https://www.mdpi.com/article/10.3390/plants10112424/s1, Table S1: comparison of commercial kits for nucleic acid extraction and purification; Description of Figure 5 diagrams.
