# Supplementary material for: The Potential Use of Isothermal Amplification Assays for In-Field Diagnostics of Plant Pathogens
Source: Plants (Basel). 2021 Nov 10;10(11):2424. doi: 10.3390/plants10112424 (PMC8621059; doi:10.3390/plants10112424)
Supplement: Supplementary file 1 [file plants-10-02424-s001.zip › plants-1389808-supplementary.pdf]

Supplementary Materials

The Potential Use of Isothermal Amplification Assays for In-Field Diagnostics of Plant Pathogens

Aleksandr V. Ivanov, Irina V. Safenkova, Anatoly V. Zherdev, Boris B. Dzantiev*

A.N. Bach Institute of Biochemistry, Research Centre of Biotechnology of the Russian Academy of Sciences, Leninsky Prospect 33, 119071 Moscow, Russia; a.ivanov@fbras.ru (A.V.I.), safenkova@inbi.ras.ru, (I.V.S.), zherdev@inbi.ras.ru (A.V.Z.), dzantiev@inbi.ras.ru (B.B.D.)

*****Correspondence: dzantiev@inbi.ras.ru; Tel.: +74959543142

CONTENT

Section S1. Comparison of commercial kits for nucleic acid extraction and purification 2

Section S2. Description of Figure 5 diagrams. 4

Section S1. Comparison of commercial kits for nucleic acid extraction and purification.

Table S1. Comparison of commercial kits for nucleic acid extraction and purification (Listed alphabetically by kit name).

Name	Manufacturer	Extraction duration (min)	Homogenization / lysis type	Separation	Capacity, µg per extraction	Thermal requirements	Equipment
AmplifyRP® Acceler8™ for TCDVd*	Adgia (USA)	<5	Mechanical (mesh)	No	ND	Room temp.	No
DNAsecure Plant Kit	TianGen® (China)	20	Grinding of frozen tissue/ND (no organic solvents, protease K added)	Column	3–30	Liquid nitrogen for homogenization, then room temp.	Centrifuge, vortex, grinder
DNeasy Blood & Tissue Kits*	Qiagen (Germany)	20–60	Mechanical (bead mill)/ND (no organic solvents, protease K added)	Silica column	6–30	Room temp.	Centrifuge, vortex
DNeasy Plant Pro and Plant Kits*	Qiagen (Germany)	<60	Mechanical by tungsten carbide beads grinding/ND (no organic solvents)	Silica column	Up to 30	65°C for lysis	TissueLyser, centrifuge, vortex, heat block
E.Z.N.A.® HP Fungal DNA Kit	Omega Bio-tek (USA)	Approx. 40	Mechanical grinding of dried or frozen tissue/ND (use chloroform and isoamyl alcohol)	HiBind® DNA Mini Column	1–24	Liquid nitrogen/oven 45°C for homogenization, extraction: up to 65°C	Kontes pellet pestle, centrifuge, vortex, heat block
FastDNA SPIN Kit for Soil*	MP Biomedicals (USA)	<30	Mechanical, sonication, enzymatic/ND	Spin column	ND	Room temp.	FastPrep® instrument for homogenization, vortex, centrifuge
Fisher BioReagents™ SurePrep™ Plant/Fungi Total RNA Purification Kit*	Thermo Fisher Scientific (USA)	30	Mechanical/ND (no organic solvents)	Spin column	30–60	Room temp.	Centrifuge, mortar and pestle, vortex
Gene-Spin™ Genomic DNA Isolation Kit	Protech Technology (Taiwan)	120–180	Mechanical grinding of frozen tissue/ND (no organic solvent, protease K added)	Spin column	ND	Liquid nitrogen for homogenization, extraction: up to 56°C	Homogenizer/ mortar and pestle, heat block
innuPREP MP Basic Kit A*	Analytik Jena (Germany)	20	Enzymatic (protease K)/ND (no organic solvents)	Magnetic beads	ND	Extraction: up to 50°	Centrifuge, vortex, heat block
MagPure Fungal DNA TL Kit	AngenBiotech (China)	60	ND/ND	Magnetic	ND	ND	ND
Monarch® Total RNA Miniprep Kit*	NEB (USA)	Approx. 20	Mechanical grinding/ND (no organic solvents)	Spin column	100	Extraction: up to 55°	Centrifuge, vortex, heat block, homogenizer
NucleoSpin Plant II, Mini kit for DNA from plants*	Macherey-Nagel (Germany)	30	Mechanical grinding/CTAB – SDS lysis (RNAse A added)	Silica column	1–30	Extraction: up to 65°	Homogenizer/ mortar and pestle, heat block
PureLink™ Plant RNA Reagent	Invitrogen (USA)	60	Mechanical grinding of frozen tissue/salt extraction with 5M NaCl with chloroform	Isopropanol precipitation	ND	Liquid nitrogen for homogenization, extraction: room temp. with 4°C cooling during centrifugation	Mortar and pestle, vortex, centrifuge with cooling
RNAiso Plus (Total RNA extraction reagent)	Takara Bio Inc (Republic of Korea)	60	Mechanical grinding of frozen tissue/ND (chloroform extraction)	Isopropanol precipitation	1000	Liquid nitrogen for homogenization,	Mortar and pestle, vortex, centrifuge with cooling

						Extraction: room temp. with 4°C cooling during centrifugation	
RNeasy Plant Mini Kit	Qiagen (Germany)	<20	Mechanical grinding of frozen tissue/ND (no organic solvents)	Spin column	25–60	Liquid nitrogen for homogenization, Extraction: room temp with 4°C during centrifugation	Mortar and pestle, vortex, centrifuge
SureFood PREP Advanced Kit*	R-Biopharm (Germany)	80	Thermal and enzymatic (protease K added)/ND (no organic solvents)	Spin column	ND	Lysis and homogenization at 65°C, then room temp.	Heat block, centrifuge, vortex
Syntol Phytosorb*	Syntol (Russia)	30	Mechanical and thermal/ND (no organic solvents)	Magnetic beads	ND	Lysis 65°C, then room temp.	Heat block, vortex, centrifuge, mortar and pestle
TRIzol™ Reagent	Invitrogen (USA)	60	Mechanical/phenol and guanidine chloride extraction	Isopropanol precipitation	ND	Room temp., cooling 4°C	Homogenizer, centrifuge with cooling, vortex
Wizard® Genomic DNA Purification Kit	Promega (USA)	90	Mechanical grinding of frozen tissue/ND (no organic solvents), thermal lysis	Isopropanol precipitation	7–12	Homogenization in liquid nitrogen, Lysis at 65°C, room temp.	Homogenizer/ mortar and pestle, heat block, centrifuge, vortex
Wizard® Magnetic DNA Purification System for Food*ǂ	Promega (USA)	40–60	ND/ND (no organic solvents)	Magnetic beads	ND	Drying at 65°C, then room temp	Heat block, centrifuge, vortex

ND – no data.
* - Kits that convenient for in-filed extraction
.

Section S2. Description of Figure 5 diagrams.

We proposed the diagrams on Fig. 5 as our interpretation of the isothermal amplification based on analysis of publication. We set PCR as the standard for all described parameters. We evaluated the parameters based on many researches data. Most of the parameters were described in the manuscript. Some parameters are quite subjective, such as "easy in field." Here we evaluated not only the number of published papers about in-field detection but our vision of the methods. For the parameter "high sensitivity," we set value 1—the method at least 100-fold less sensitive than PCR, value 2—the method 10–100-fold less sensitive than PCR, or value 3—the method no more than 10-fold less sensitive or more sensitive than PCR. For "high specificity" value 1, the method cannot discriminate more than three mismatches, and for value 3, the method is able to discriminate no more single mismatches. Also, we made some speculations according to the temperature of primer annealing. The higher the temperature, the higher the specificity. We declare parameter "minimal equipment" 3 for all isothermal methods by the nature of assays. "Tolerance to inhibition" is quite speculative because few papers were published where clear results were presented. However, based on the possibility of RPA and LAMP proceed in crude extract, we concluded that they obtain high tolerance to plant inhibitors. Values for parameter "rapid" were set as 1—the method completed within 30 min, 2—the method completed within 30–60 min, or 3—the method completed in more than 60 min. The parameter "multiplexity" was estimated as the common possibility to have multiplexing and the number of papers describing the multiplexes.
